# A new maturity recognition algorithm for Xinhui citrus based on improved YOLOv8

**DOI:** 10.3389/fpls.2025.1472230

**Published:** 2025-01-29

**Authors:** Fuqin Deng, Zhenghong He, Lanhui Fu, Jianle Chen, Nannan Li, Weibiao Chen, Jialong Luo, Weilai Qiao, Jianfeng Hou, Yongkang Lu

**Affiliations:** ^1^ School of Electronic and Information Engineering, the Wuyi University, Jiangmen, China; ^2^ School of Computer Science and Engineering, Faculty of Innovation Engineering, Macau University of Science and Technology, Macao, Macao SAR, China; ^3^ School of Mechanical and Automation Engineering, The Wuyi University, Jiangmen, China; ^4^ College of Advanced Engineering, Great Bay University, Dongguan, China

**Keywords:** object detection, maturity detection, XinHui citrus, YOLOv8, CARAFE lightweight operator, multi-dimensional collaborative attention mechanism (MCA), GhostConv

## Abstract

Current object detection algorithms lack accuracy in detecting citrus maturity color, and feature extraction needs improvement. In automated harvesting, accurate maturity detection reduces waste caused by incorrect evaluations. To address this issue, this study proposes an improved YOLOv8-based method for detecting Xinhui citrus maturity. GhostConv was introduced to replace the ordinary convolution in the Head of YOLOv8, reducing the number of parameters in the model and enhancing detection accuracy. The CARAFE (Content-Aware Reassembly of Features) upsampling operator was used to replace the conventional upsampling operation, retaining more details through feature reorganization and expansion. Additionally, the MCA (Multidimensional Collaborative Attention) mechanism was introduced to focus on capturing the local feature interactions between feature mapping channels, enabling the model to more accurately extract detailed features, thus further improving the accuracy of citrus color identification. Experimental results show that the precision, recall, and average precision of the improved YOLOv8 on the test set are 88.6%, 93.1%, and 93.4%, respectively. Compared to the original model, the improved YOLOv8 achieved increases of 16.5%, 20.2%, and 14.7%, respectively, and the parameter volume was reduced by 0.57%. This paper aims to improve the model for detecting Xinhui citrus maturity in complex orchards, supporting automated fruit-picking systems.

## 1 Introduction

Xinhui dried tangerine peel(Chenpi), regarded as the finest among Guangdong dried tangerine peels, is a traditional authentic Chinese medicinal material. It is considered one of the three treasures of Guangdong and is also listed among the top ten medicinal materials (Lin et al., 2009). The expected total output value of dried tangerine peel in 2023 is 23 billion yuan. And Xinhui citrus is the raw material for making tangerine peel. Mechanical picking has become a focal point of research in recent years, with recognition being the foundation for successful harvesting (Zheng et al., 2021). Enhancing recognition accuracy can significantly reduce the incidence of mechanically picking non-target citrus fruits (Li et al., 2021). In actual production, citrus fruits of varying maturities have different economic values. Additionally, identifying the ripening stage of citrus fruits directly affects their transportation and storage methods. People usually judge citrus maturity by observing the skin color. While this intuitive method meets daily needs, it is insufficient for large-scale mechanized picking (Zheng et al., 2023) and tangerine peel production. Therefore, studying an automatic maturity detection system with a high recognition rate is of great significance for promoting the automatic picking (Zheng et al., 2024b) of Xinhui citrus.

In recent years, to promote the development of intelligent agriculture (Ying et al., 2004), domestic and international scholars have explored using spectral analysis and machine vision methods to detect the maturity of various fruits, such as navel oranges (Wei et al., 2017), banana (Zheng et al., 2024a) and sweet peppers (Castro et al., 2019). Yuan et al. (2023) used spectral methods to distinguish different maturity stages of camellia oleifera fruit, but this process is prone to generating a large amount of noise and interference. Zou (2023) studied the use of machine vision technology to identify the external color of citrus fruits and to determine their color grade. Although this method was implemented in a laboratory detection environment after harvesting, it provides a valuable technical reference for the application of automatic picking technology in complex natural environments. Zhou et al. (2020) proposed a maturity detection method for red grapes based on an improved circular Hough transform, providing theoretical guidance for achieving automated picking. The machine learning methods mentioned above often rely on manual feature extraction, which tends to have poor robustness in complex scenarios and struggles with real-time maturity detection under natural conditions (Lv et al., 2019). Consequently, some researchers have begun to use deep learning methods for fruit maturity recognition and classification.

With advancements in computer system performance and computing power, deep learning algorithms have become widely used for identification and detection in agricultural fields (Song et al., 2023). Due to the significant advantages of deep learning technology in agriculture, including its ability to perform detection tasks quickly and accurately, an increasing number of scholars are integrating computer vision with agriculture (Tang et al., 2024). This integration is being applied to various production stages, such as crop cultivation (Bi et al., 2019), harvesting (Fu et al., 2022) and other agricultural processes (Lv et al., 2019). Due to the characteristic of fruits having inconsistent ripening periods during growth, harvesting robots need to determine the ripeness of the fruits before issuing picking commands (Chen et al., 2024). Therefore, harvesting robots need to be equipped with high-precision recognition systems (Momeny et al., 2022). Azarmdel et al. (2020) applied artificial neural networks (ANN) and support vector machines (SVM) to classify the maturity of mulberry fruits, achieving detection and classification accuracy of 98.26%. Harvesting robots’ embedded devices require faster detection algorithm models (Zhang et al., 2024). The YOLO algorithm (Redmon et al., 2016), known for its ability to quickly and accurately detect different objects, has been widely applied in the agricultural field (Wang et al., 2024). Xiong et al. (2023) improved YOLOv5 by using MobilenetV3 as the backbone feature extraction network and replacing conventional convolution with depthwise separable convolution. They achieved an average precision of 92.4% in recognizing the maturity of papaya fruits in natural environments. Chen et al. (2023) improved the YOLOv5s backbone network and incorporated a full-dimensional dynamic convolution module into the neck structure. This modification successfully enabled the detection of strawberry fruit maturity in greenhouse environments, achieving an average precision of 97.4%. Miao et al. (2023) incorporated MobileNetV3 into YOLOv7 and added a global attention mechanism to the feature fusion network. This enhancement improved the detection accuracy of fruits with adjacent maturity stages and occluded fruits, achieving an average precision of 98.2%. Yang et al. (2023) incorporated the Swin Transformer structure, which offers improved feature extraction capabilities, resulting in an increase in average precision. Ni et al. (2024) proposed a lightweight YOLOv8 model that introduced a reconstruction convolution module, achieving an average precision of 98.1%. The above methods have achieved significant success in agricultural object detection, strongly supporting the realization of intelligent harvesting.

The detection accuracy and the number of parameters in a model are crucial for its application in target detection on smart agriculture mobile devices (Liu et al., 2024). Other models exhibit limited capability in extracting color features of citrus fruits at different maturity levels, which adversely affects classification accuracy. At the same time, under natural conditions, the same fruit tree can have fruits at different maturity stages, and not all fruits may meet the harvesting standards. Therefore, accurately assessing fruit maturity helps to reduce waste caused by harvesting immature fruits. This paper proposes an improved YOLOv8 model for detecting the maturity of Xinhui citrus. First, during datasets construction, images of citrus fruits at different maturity stages are collected. Based on the YOLOv8 model, GhostConv (Han et al., 2020) is used to replace some of the conventional convolutions in the Head, reducing the number of parameters and improving parameter deployment. This approach helps to lower the complexity of convolutional computations. Next, the upsampling module of YOLOv8 is improved by replacing the transpose convolution-based upsampling operation with the CARAFE upsampling operator (Wang et al., 2019). This modification, which involves feature reorganization and expansion, retains more detailed information and enhances the model’s detection accuracy. Additionally, the MCA attention mechanism (Yu et al., 2023) is introduced to capture local feature interactions between feature mapping channels, helping the model to more accurately extract and understand detailed features. This further improves the accuracy of citrus maturity recognition.

The main contributions of this study are as follows:

1. Model Innovation: This study introduces an improved YOLOv8 model specifically designed for detecting the maturity of Xinhui citrus in complex backgrounds. Through meticulous optimization of the network architecture and the integration of advanced attention mechanisms, this model achieves outstanding accuracy even in challenging scenarios.
2. Dataset Development: This study constructs a comprehensive dataset comprising real citrus fruit images captured in orchard environments. This dataset serves as a valuable resource for training and evaluating the model, providing diverse and realistic data to achieve optimal performance assessment.
3. Performance Enhancement: This study leverages the combination of CARAFE, GhostConv, and MCA attention mechanisms to enhance color information recognition, significantly improving detection accuracy and computational efficiency.
4. Real-Time Application Potential: This study’s method features a compact model size and exceptional computational efficiency, making it a viable solution for real-time citrus fruit detection applications. This technological advancement greatly supports intelligent management in citrus orchards and ensures a steady supply of raw materials for citrus peel production.

## 2 Dataset construction

### 2.1 Image acquisition

**Figure 1:**
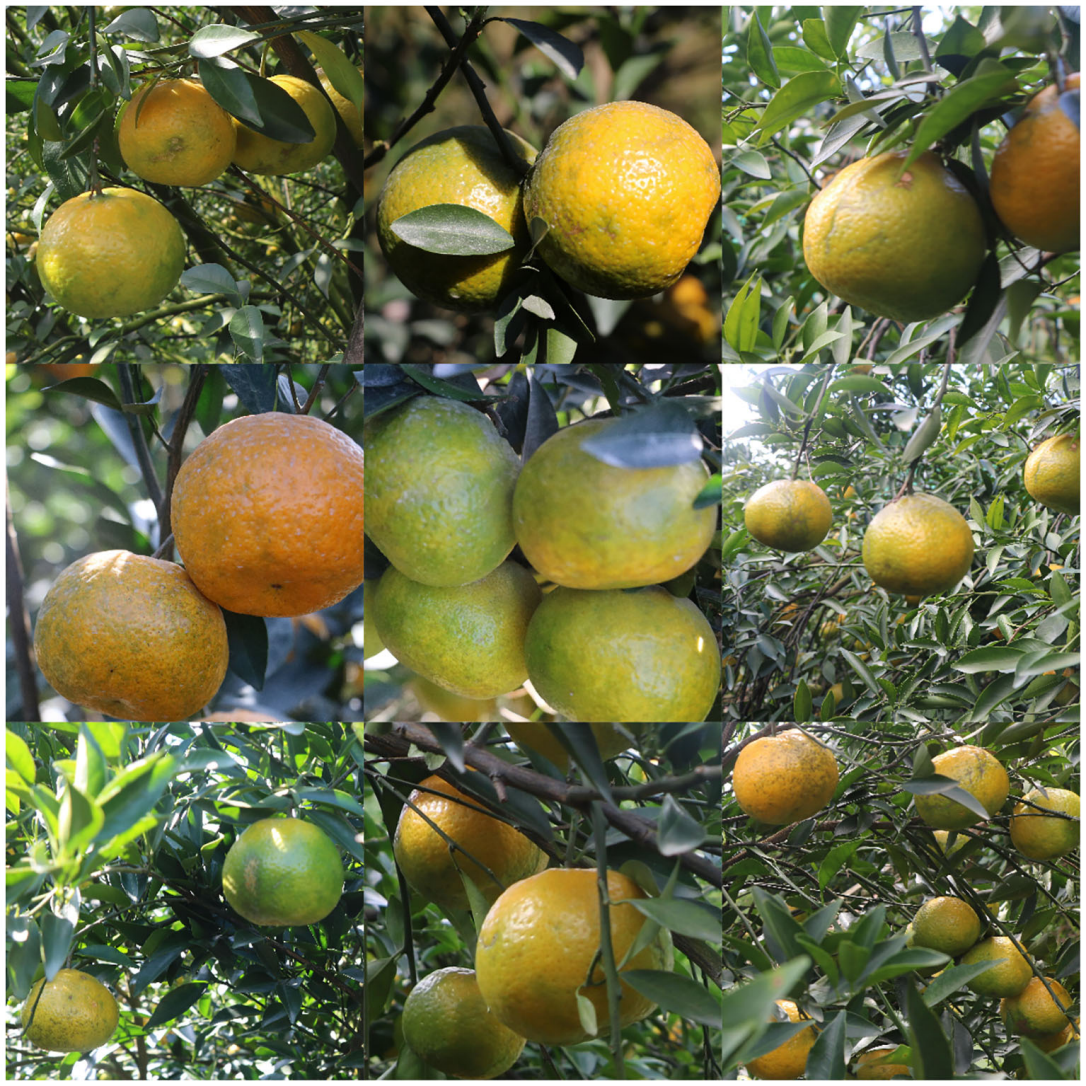
Sample images were collected from Xinhui citrus planting.

The experimental research area selected was the Xinhui citrus planting base in Dongjia Village, Xinhui District, Jiangmen City, Guangdong Province. The main cultivars in this base are Xinhui citrus, Emperor citrus, and Wogan citrus. This paper focuses on Xinhui citrus, which has the highest yield and the greatest economic and medicinal value. Images of Xinhui citrus at different maturity stages were collected using a Canon 760D SLR camera under natural environmental conditions from October to December 2023. The image acquisition times included noon and afternoon to capture images under varying lighting conditions. A total of 1793 images of Xinhui citrus with 6000 × 4000 pixels resolution were obtained. The complex orchard environment, shown in **Figure 1**, includes various lighting conditions such as direct light and backlight, as well as different scenarios such as single fruit, multiple fruits, close-up views, distant views, fruit overlap, and occlusion by branches and leaves.

**Figure 2:**
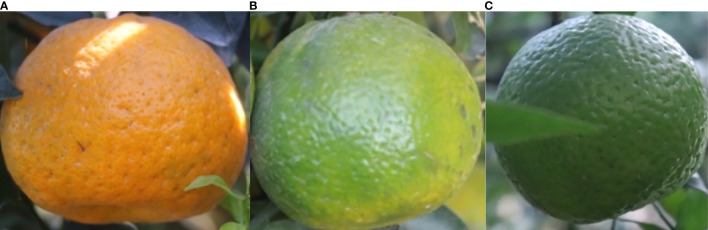
images of different maturity levels Citrus. **(A)** Mature XinHui citrus. **(B)** Semi-mature XinHui citrus. **(C)** Immature Xinhui citrus.

When labeling, citrus fruits are categorized into three types: semi-ripe fruits (yellow-green), fully ripe fruits (fully orange peel), and unripe fruits (green), as shown in **Figure 2**. The labeled dataset is divided into training, testing, and validation sets in a random 8:1:1 ratio, resulting in 1656, 64, and 73 images, respectively.

### 2.2 Fruit maturity grade division

The maturity of Xinhui citrus is divided into three stages in the market: green citrus, Erhong citrus(middle red), and bright red citrus. Xinhui citrus at different maturity stages has varying demands and prices in the market. The dried peel of citrus fruits at different maturity stages also differs in appearance, taste, and use. Green citrus has a thin skin, is resistant to storage, and has a green appearance. It is suitable for long-distance transportation and storage, making it ideal for producing green citrus peel or green citrus tea. Erhong citrus is an immature citrus fruit that is relatively resistant to storage. It has a hard texture, thick skin, and a green-yellow appearance, making it suitable for artificial ripening, long-distance fresh market sales, or as raw material for producing Erhong citrus peel. Bright red citrus is a fully ripe fruit that is not easy to store, making it suitable for direct consumption or production into tangerine peel. It has a soft, thick skin and an orange-yellow appearance.

### 2.3 Data set construction

The datasets used for model training in this study follows the YOLO series format. Xinhui citrus fruits at different maturity stages in the images were annotated using LabelImg software. The annotation rules for the txt. documents are as follows: (1) Fruits in the annotated image can block each other as long as it does not affect the manual judgment of maturity. (2) Severely blocked fruits should not be annotated.

## 3 The maturity detection model of Xinhui citrus

In this study, the YOLOv8n model was improved to balance detection speed, accuracy, and computational complexity, and to better address the detection of fruits with adjacent maturity stages. The YOLOv8 network structure consists of three main components: Backbone, Neck, and Head. The Backbone is responsible for extracting initial features from the input image, transforming the original image into feature maps rich in information, and providing the basis for subsequent feature fusion and target detection.

**Figure 3:**
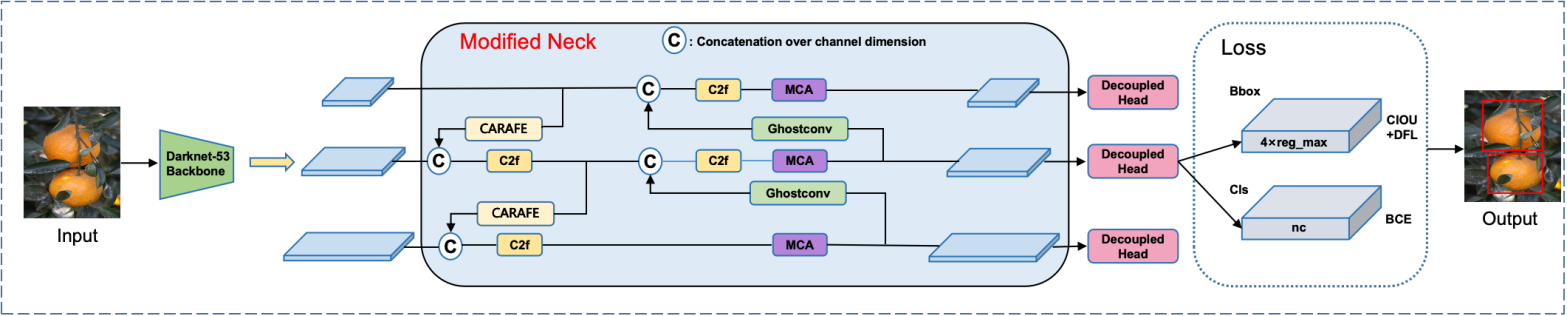
YOLOv8 Network structure diagram of the Xinhui citrus maturity detection model.

Improvements were made to the Neck part of the YOLOv8 model. The network structure diagram of the improved YOLOv8 Xinhui citrus maturity detection model is shown in **Figure 3**. To achieve fast detection speed while maintaining high accuracy and reducing model computational parameters, GhostConv was used to replace some conventional convolutions in the network structure. To overcome the limitations of the original upsampling, the lightweight upsampling operator CARAFE (Content-Aware Reassembly of Features) was introduced. CARAFE allows the model to dynamically adjust the upsampling process based on the content of different parts of the feature map, effectively utilizing contextual information. This makes the model more precise in handling citrus color and texture, thereby better distinguishing citrus fruits with different maturities.

Additionally, to enhance the network model’s feature extraction capability for citrus, the MCA attention mechanism (Multidimensional Collaborative Attention) was added to the network. This effectively suppresses the interference of non-target background information, further improving the model’s accuracy in detecting citrus maturity.

### 3.1 CARAFE lightweight up sampling operator

The upsampling operation in the YOLOv8 algorithm is implemented using nearest neighbor interpolation, which performs a convolution operation on the input feature map to enlarge its size and increase resolution. However, when processing large-sized images, upsampling operations often require substantial computational resources, and nearest neighbor interpolation can cause discontinuity in images or data, leading to information loss and affecting model performance.

To address this issue, this paper introduces the lightweight and efficient CARAFE operator in YOLOv8 to optimize the upsampling operation. The CARAFE (Content-Aware Reassembly of Features) operator is a lightweight upsampling method that better retains the information in the feature map and reduces information loss. Unlike the upsampling in YOLOv8, the CARAFE operator not only considers the nearest neighbor points but also combines the content information of the feature map to achieve more accurate upsampling by recombining the feature map information.

**Figure 4:**
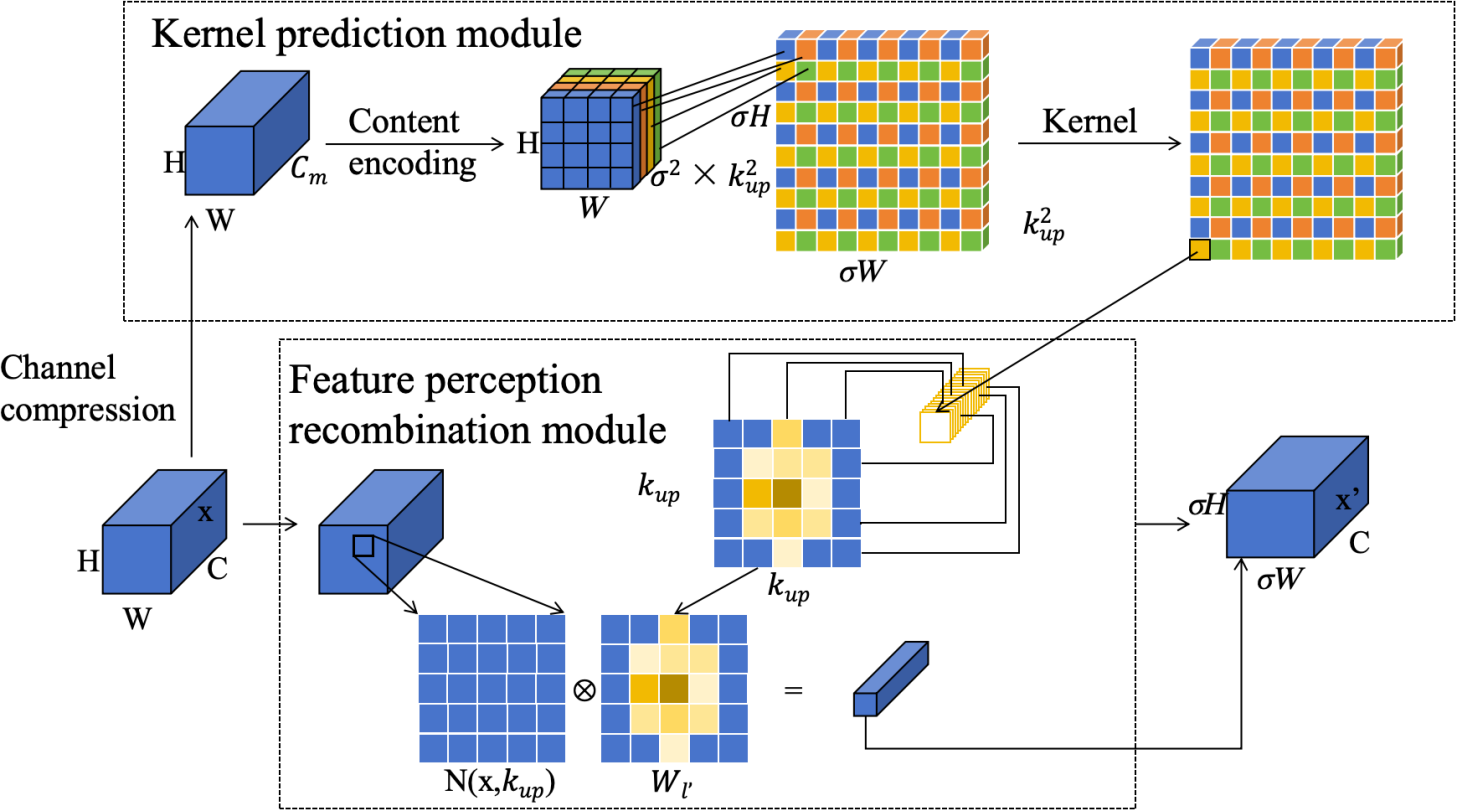
CARAFE lightweight upsampling operator structure diagram.

This enables the CARAFE operator to better process large-size images, reduce computational resource consumption, and maintain the continuity of feature maps more effectively. Consequently, the model’s ability to detect and classify the maturity of Xinhui citrus is significantly improved. The CARAFE operator consists of the Kernel Prediction Module and the Content-aware Reassembly Module. The structure is shown in **Figure 4**. In the kernel prediction module, the H×W×C input feature map is subjected for channel compression. The compressed feature map is Content encoder through the convolution kernel of $k_{up}\times k_{up}$ , and the recombination kernel is generated to obtain the feature map of $\sigma^{2}\times k_{up}^{2}$ , where is the upper sampling rate. Then, the channels are expanded in the spatial dimension, and then arranged and combined according to the law to obtain the $k_{up}^{2}\times \sigma \text{H}\times \sigma w$ up-sampling kernel. In order to reduce the amount of computation, Softmax normalization is performed on the upper sampled kernel, so that the sum of the weights of the convolution kernel is 1. In the feature sensing recombination module, each position in the output feature map is mapped back to the input feature map and a region of size $k_{up}\times k_{up}$ is taken with the target as the center. The dot product operation is done with the upper sampling kernel obtained by the prediction of this point, and the feature map of  σH×σW×C is obtained.

Therefore, CARAFE improves feature upsampling in YOLO by reorganizing features through a content-aware mechanism, enhancing feature map resolution and semantic information. This reorganization helps YOLO achieve more accurate target recognition and localization, particularly improving detection precision and color feature recognition.

### 3.2 The GhostConv module

In classical feature extraction methods, multiple convolution kernels are used to represent each path of the input feature map. This approach relies on a large number of parameters, leading to the generation of many redundant feature maps, which reduces the efficiency of deep learning and makes it difficult to ensure the accuracy of the feature maps. To address these issues, this paper integrates the GhostConv module into the YOLOv8 network. By dividing the convolution operation into two stages, the main convolution extracts key features, and GhostConv reduces the number of parameters and computation. This approach improves efficiency and processes redundant feature mapping more effectively.

**Figure 5:**
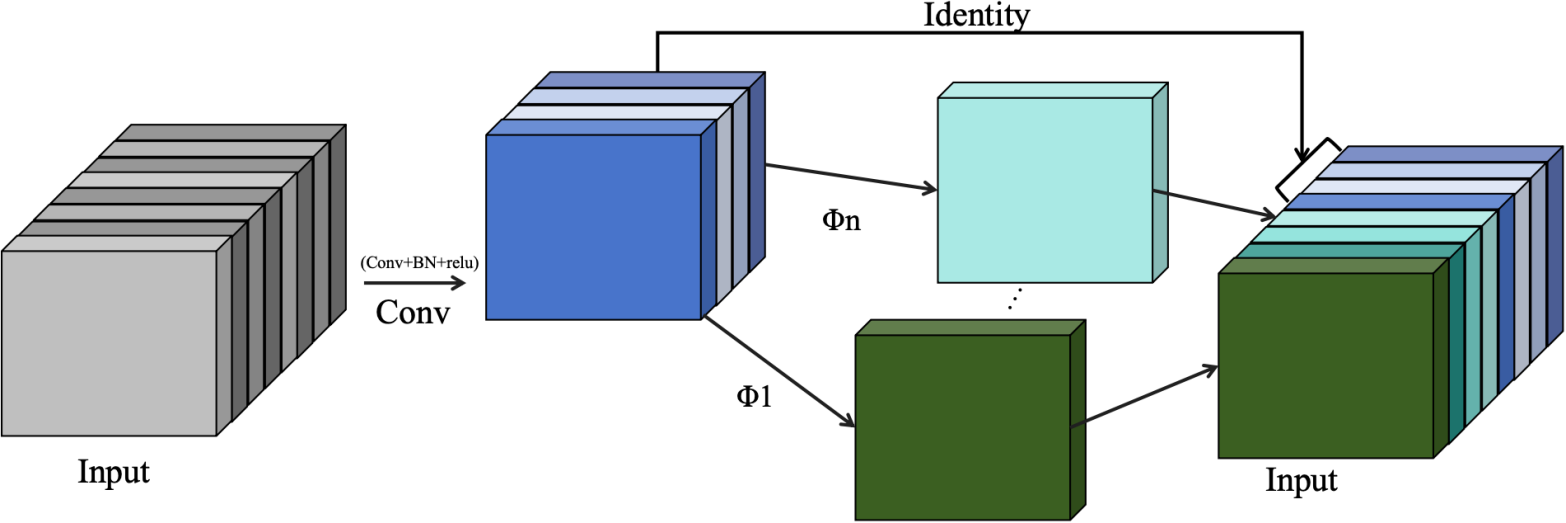
GhostConv module structure diagram.

The GhostConv module reduces the computation and parameters of the network while maintaining the original channel size and the size of the convolution output feature map. The structure of the extracted features is shown in **Figure 5**. The GhostConv module consists of three parts: the input part extracts the input feature map through the convolution of 1 × 1 to obtain the feature map Y; the middle part calculates the single channel through deep convolution (Depth-wise convolutional); the output part combines the feature map (blue) of the first part and the second redundant feature map (green) (Concat) to obtain the output feature map. This feature graph shows the number of n-dimensional channels, where Φ1…Φn Characteristic graph of different dimensions and Identity for identity mapping. And h’ is the output feature height; w’ is the output feature width; c is the number of input channels; and g is the conventional convolution kernel size.

In ordinary convolution, the formula is:


(1) $$Flop_{conv}=n\times h^{\prime }\times w^{\prime }\times c\times g\times g$$

Suppose the size of the convolution kernel of a linear transformation operation is r×r , then each basic feature corresponds to a feature redundancy, and the number of s is less than the number of channels. The common convolution method obtains m feature graphs, and the transformation process of Ghost module has identity transformation, and the calculation amount of Ghost module is as follows:


(2) $$\begin{array}{c} Flop_{Ghostconv}=\frac{n}{s}\times h^{\prime }\times w^{\prime }\times g\times g+\left(s-1\right)\times \frac{n}{s}\\ \;\;\;\times h^{\prime }\times w^{\prime }\times r\times r \end{array}$$

The computational load of GhostConv compared to standard convolution is as follows:


(3) $$\begin{array}{c} \frac{Flop_{conv}}{Flop_{Ghostconv}}=\frac{n\times h^{\prime }\times w^{\prime }\times c\times g\times g}{\frac{n}{s}\times h^{\prime }\times w^{\prime }\times g\times g+\left(s-1\right)\times \frac{n}{s}\times h^{\prime }\times w^{\prime }\times r\times r}\\ =\frac{c\times g\times g}{\frac{1}{s}\times c\times g\times g+\frac{s-1}{s}\times r\times r}\approx \frac{s\times c}{s+c-1}\approx s \end{array}$$

Therefore, the computational load of standard convolution is approximately s times that of the Ghost module.

In summary, the Ghost module in the YOLO framework enhances feature map generation efficiency and optimizes feature representation. By splitting the convolution layer into two parts, it generates a few intrinsic feature maps with limited filters and produces additional “ghost” feature maps through low-cost linear transformations. This reduces parameters and computational complexity while improving feature expression. In YOLO, it maintains high detection accuracy, reduces computational load, accelerates inference, and improves performance in detecting small objects and dense scenes, making it suitable for resource-constrained devices.

### 3.3 Multidimensional collaborative attention mechanism MCA

**Figure 6:**
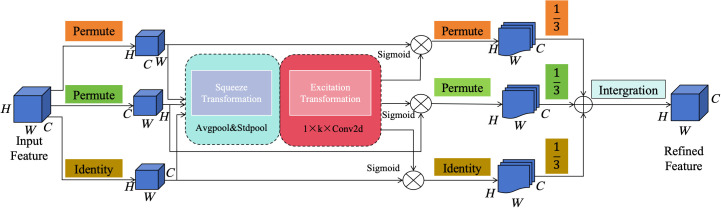
Shows the overall architecture of the MCA module, which includes three branches. The symbol ⊗ represents the element wise multiplication of broadcasting, and ⊕ represents the element wise sum of broadcasting.

The Multidimensional Collaborative Attention (MCA) mechanism is an efficient attention mechanism designed to address issues in existing fruit detection methods, such as ignoring attention modeling in the channel and spatial dimensions, and increasing model complexity and computational load. This paper introduces the MCA method to optimize these aspects. The MCA module comprises three components: the Squeeze mechanism, the Excitation mechanism, and Integration. Its structure is shown in **Figure 6**.

The Squeeze mechanism effectively combines average pooling and standard deviation pooling. The Excitation mechanism adaptively determines the interaction coverage to obtain local feature interactions between channels. During the Integration phase, the three branches converge, generating a refined feature map. This method employs a three-branched architecture to simultaneously deduce channel, height, and dimensional attention without additional computational consumption.

The top branch captures interactions between features in spatial dimensions, the middle branch also addresses interactions in spatial dimensions, and the bottom branch captures interactions between channels. In the first two branches, a permutation operation is used to capture the long-range correlation between the channel dimension and any one of the spatial dimensions. Finally, the outputs of all three branches are aggregated through simple averaging during the Integration phase.

Therefore, the design of MCA enables the model to adaptively aggregate feature responses across dimensions and effectively capture local feature interactions, enhancing the model’s recognition accuracy. Moreover, as a module, MCA introduces minimal computational overhead, allowing it to be integrated into various CNN architectures without compromising inference speed. Consequently, MCA improves the quality of feature representation and computational efficiency of the model, resulting in an increase in mAP for image recognition tasks.

### 3.4 Evaluation index of neural network model

The detection process of Xinhui citrus maturity needs to consider both the detection accuracy and the computational complexity of the model. To evaluate model detection accuracy, we use Precision (P) and Recall (R) are shown in Equations 4, 5. Average Precision (AP) as evaluation indicators. For assessing model detection performance, Mean Average Precision (mAP) is used. The formulas for Precision (P) and Recall (R) are shown below, where true positive (TP), false positive (FP), true negative (TN), and false negative (FN) are used:


(4) $$\begin{array}{c} P=\frac{TP}{TP+FP}\; \end{array}$$

(5) $$\begin{array}{c} R=\frac{TP}{TP+FN} \end{array}$$

AP (Average Precision) is an indicator used to measure the detection accuracy of the model. It reflects the average performance accuracy across different categories by calculating the area under the Precision-Recall (P-R) curve. The calculation formula for Average Precision (AP) is shown in Equation 6:


(6) $$\begin{array}{c} AP=\int_{0}^{1}\left(pre\times rec\right)drec \end{array}$$

The mAP (mean Average Precision) is the average of all categories of AP, obtained by summing and averaging each AP value, The calculation formula is shown in Equation 7:


(7) $$\begin{array}{c} mAP=\frac{sum\left(AP\right)}{n} \end{array}$$

## 4 Experimental results and analysis

### 4.1 Experimental environment configuration

The operating system runs on Linux, with a Core i9-9900k CPU and an NVIDIA GeForce RTX 3090 GPU. It has 24 GB of RAM and a 1 TB mechanical hard disk. The programming language used is Python 3.6, and the deep learning framework is PyTorch version 1.13.1 with CUDA version 11.7. To optimize training efficiency and achieve the best training weights, Patience was set to 30, the batch size was set to 16, the epoch was set to 150, the optimizer were set to AdamW and the number of workers was set to 4.

### 4.2 Xinhui citrus maturity detection experiment

To validate the performance of the improved YOLOv8 model, this study evaluated a test set of Xinhui citrus samples at different maturity stages. **Table 1** presents the detection results of the improved YOLOv8 model on these samples. According to the data in **Table 1**, the improved YOLOv8 model achieved an average precision (mAP) of 93.4%, with a precision (P) of 88.6% and a recall (R) of 93.1%.

**Table 1:** The detection results of improved YOLOv8 model on different maturity levels of Xinhui citrus.

Maturity stages	Precision	Recall	mAP_50_	mAP_50-95_
immature	1	0.943	0.991	0.884
fully mature	0.873	0.87	0.949	0.881
semi-mature	0.786	0.98	0.862	0.737
average value	0.886	0.931	0.934	0.834

The improved YOLOv8 model detected 100% of the immature Xinhui citrus, demonstrating its effectiveness in distinguishing the fruit from the background. However, for the semi-mature and fully mature Xinhui citrus, the model exhibited a tendency to misjudge the colors of semi-mature and fully mature citrus in the datasets. To address this issue, a multi-dimensional collaborative attention (MCA) module was introduced in the YOLOv8 model. This module captures feature interdependencies in the spatial dimension from three branches, enhancing the model’s feature extraction capability for semi-mature Xinhui citrus. Consequently, the detection performance of the improved YOLOv8 model has significantly increased compared to the original YOLOv8 model. The specific improvement in detection results can be seen in the ablation experiment data.

**Figure 7:**
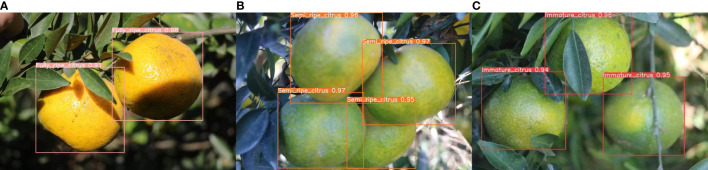
Maturity detection result chart. **(A)** Mature XinHui citrus. **(B)** Semi-mature XinHui citrus. **(C)** Immature Xinhui citrus.

**Figure 7** shows part of the detection results, illustrating the model’s ability to perform the maturity detection task even with slight occlusions in the citrus images. The improved algorithm incorporates positional and semantic information of occluded fruits, enabling the model to accurately detect the maturity of fruits blocked by leaves. In conclusion, the improved YOLOv8 model can reliably and accurately detect fruit maturity.

### 4.3 Improved YOLOv8 model ablation experiments

To further validate the performance of the improved YOLOv8 model, this study designed ablation experiments designed to validate the performance of the 8 sets of models. Based on YOLOv8, MCA attention module is introduced, GhostConv is used to replace the standard convolution in YOLOv8, and CARAFE is used to replace the upsampling of YOLOv8. The performance of the 8 networks was analyzed from a quantitative perspective and objectively evaluated by the test set, and the comparison results are shown in **Table 2**. Among them, Experiment 1 is the basic network YOLOv8, while Experiment 2-8 is the network after adding or replacing various modules on the basic network.

**Table 2:** Results of ablation experiments for improving the model based on YOLOv8.

Experiment number	MCA	Ghost	CARAFE	Precision	Recall	mAP_50_	Parameter	GFLOPs
1	–	–	–	0.721	0.729	0.787	3006233	8.2
2	√	–	–	0.780	0.882	0.865	3006263	8.1
3	–	√	–	0.801	0.921	0.927	1714661	5.0
4	–	–	√	0.841	0.826	0.826	3078657	8.3
5	√	√	–	0.848	0.901	0.925	2916503	8.0
6	√	–	√	0.701	0.829	0.876	3078687	8.3
7	–	√	√	0.780	0.882	0.865	3006263	8.2
8	√	√	√	0.886	0.931	0.934	2988927	8.3

Use “√” to indicate improvement, use “-” to indicate no improvement used.

Experiment 2 used the MCA attention mechanism, which enhanced the performance of the YOLOv8 baseline model. This attention mechanism focuses on capturing local feature interactions between feature mapping channels, enabling the model to extract and understand detailed features more precisely and thereby improving overall performance. By combining global interactions between channels and local interactions between feature mapping channels, the MCA attention mechanism comprehensively captures important information in the image, significantly enhancing the quality of feature expression. This mechanism further improves the accuracy of citrus maturity identification by introducing interactive operations between different channels, allowing each channel to gather more context information from others. Experiment 3 replaced the convolution of the Head part in YOLOv8 with GhostConv, which effectively reduced the number of parameters as a lightweight convolution. Experiment 4 introduced the CARAFE upsampling operator, and although the number of parameters increased slightly, the accuracy, recall, and average precision all improved. The CARAFE upsampling operator, by replacing the original upsampling operation, retains more detailed features, reduces the impact of feature loss, lowers the leakage rate, and verifies the superiority of CARAFE in performance improvement. Experiments 5 to 7 introduced different combinations of modules to verify the compatibility of each module combination. In Experiment 8, three modules were added to YOLOv8 simultaneously, achieving the highest accuracy, recall, and average precision, with significant performance improvements, indicating that the overall comprehensive performance of the model was optimal. Relative to the YOLOv8 baseline model, the introduction of these three modules improves accuracy, recall, and average precision with a reduced number of parameters, verifying the feasibility and effectiveness of these modules on YOLOv8.

### 4.4 Improve the performance comparison test between YOLOv8 and other models

In order to further verify the effectiveness of the improved YOLOv8, this study tested the same datasets. This datasets contains a total of 1793 images of Xinhui citrus fruits. Through the YOLOv8 original model, YOLOv7 (Wang et al., 2023) and YOLOv9 (Wang et al., 2025) respectively, and the experimental results are shown in **Table 3**. The improved YOLOv8 has the highest mAP value at 93.4%. YOLOv7 has the lowest index. YOLOv9 is the latest algorithm of YOLO series. YOLOv9 incorporates a deeper network structure and integrates Transformer modules. As a result, the model performs poorly on small datasets or standard hardware environments, as overly deep or complex networks are prone to overfitting or reduced inference efficiency. Additionally, YOLOv9 may require more computational resources to achieve its accuracy advantages.

**Table 3:** Detection results of the different models.

Model	Precision	Recall	mAP_50_	mAP_50-95_	Parameters	GFLOPs
Improved YOLOv8	0.886	0.931	0.934	0.868	2988927	8.3
YOLOv8	0.721	0.729	0.787	0.656	3006233	8.2
YOLOv7	0.742	0.883	0.877	0.779	36492560	103.2
YOLOv9	0.831	0.914	0.927	0.851	60801810	266.1

**Figure 8:**
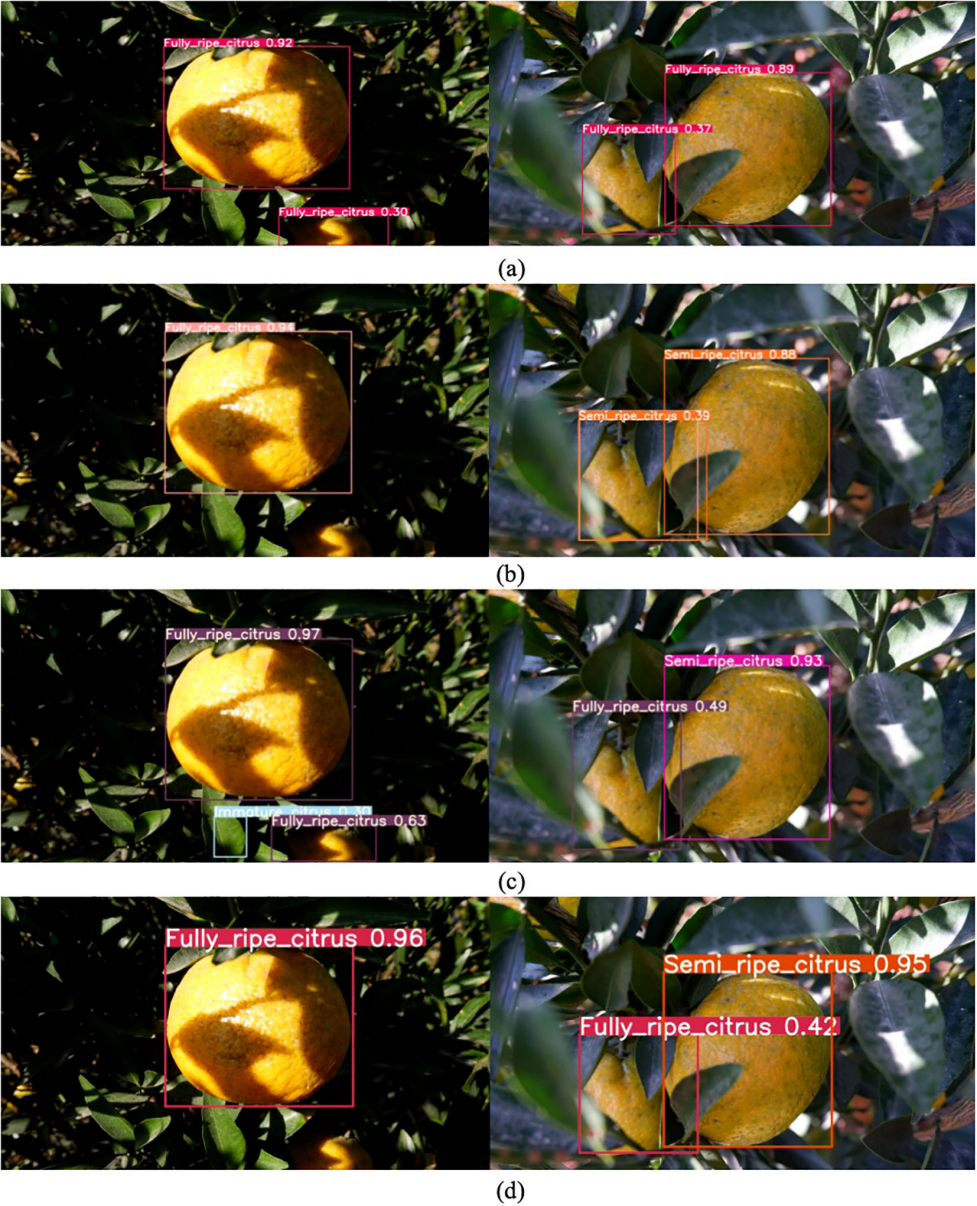
Comparison of detection results in the test set. **(A)** Improved YOLOv8; **(B)** YOLOv8; **(C)** YOLOv7; **(D)** YOLOv9.

Compared with the improved YOLOv8, all evaluation indexes are lower than the improved YOLOv8. For example, the image pair in the test set is shown in **Figure 8**. There is a total of 73 images of Xinhui citrus with different maturity levels in the test set. A comprehensive comparison of the test set reveals that, in **Figure 8** of the left panel, the detection capability of the improved YOLOv8 algorithm is significantly stronger. The YOLOv8 and YOLOv9 algorithms exhibit instances of missed detection, while YOLOv7 demonstrates misdetection. In the right panel, the improved YOLOv8 successfully completes the detection of Xinhui citrus maturity, whereas YOLOv7 and YOLOv9 misjudge the maturity. In conclusion, the improved YOLOv8 demonstrates advantages in detecting the maturity of Xinhui citrus, even under uneven leaf shading, and effectively completes the task of maturity detection.

## 5 Discussion

The improved YOLOv8 model, incorporating GhostConv, CARAFE upsampling, and the MCA attention mechanism, enhances the extraction of citrus ripeness color features. These updates improve detection accuracy, reduce model parameters, and make it suitable for mobile intelligent agricultural devices.

Deep learning methods (Xu et al., 2024) for fruit ripeness detection are widely studied and prove the importance of ripeness evaluation (Chen et al., 2023). While this study advances citrus ripeness detection, some limitations remain. Considering hardware cost constraints, real-time ripeness detection must be optimized for broader use in harvesting robot systems.

## 6 Conclusions

This paper proposes an improved YOLOv8 model for detecting the maturity of Xinhui citrus, addressing the issue of insufficient accuracy in detecting citrus ripening colors using detection algorithms. By replacing ordinary convolutions with GhostConv in the Head and using transpose convolution for upsampling, the model reduces parameters and computation complexity while enhancing detection accuracy. The MCA mechanism improves local feature interaction, further boosting accuracy. After extensive training and validation on a large dataset, the results demonstrate that the improved model achieves 88.6% precision, 93.1% recall, and 93.4% average accuracy, representing improvements of 16.5%, 20.2%, and 14.7%, respectively, along with a 0.57% reduction in error.

To verify performance, eight sets of networks were established for ablation experiments, incorporating GhostConv, MCA, and CARAFE modules into YOLOv8 in different combinations. Results show the improved YOLOv8 surpasses other models in detection accuracy, recall, and average accuracy while reducing computational load. Under the same conditions, the improved YOLOv8 outperformed YOLOv7 and YOLOv9 on Xinhui citrus datasets, with 5.5%, 1.7%, and 0.7% higher precision, recall, and average accuracy than YOLOv9, and having about 5% and 1.2% of YOLOv9’s parameters. The algorithm presented in this paper has not yet seen widespread practical deployment. Our future plans include integrating it into mobile harvesting robots to enable precise detection of citrus maturity, ensuring that only citrus meeting the desired maturity criteria are harvested. This study supports intelligent picking in smart agriculture and offers reference suggestions for future work in target detection, visual positioning, and classification in smart agriculture.

## Data Availability

The original contributions presented in the study are included in the article/supplementary material. Further inquiries can be directed to the corresponding author.
